# Emulation of synaptic functions with low voltage organic memtransistor for hardware oriented neuromorphic computing

**DOI:** 10.1038/s41598-022-07505-9

**Published:** 2022-03-09

**Authors:** Srikrishna Sagar, Kannan Udaya Mohanan, Seongjae Cho, Leszek A. Majewski, Bikas C. Das

**Affiliations:** 1 School of Physics, Indian Institute of Science Education and Research Thiruvananthapuram (IISER TVM), Vithura, Trivandrum, Kerala 695551 India; 2 Department of Electrical and Electronic Engineering, University of Manchester, Manchester, M13 9PL UK; 3 Department of IT Convergence Engineering, Gachon University, Seongnam, Republic of Korea

**Keywords:** Information storage, Electronic and spintronic devices

## Abstract

Here, various synaptic functions and neural network simulation based pattern-recognition using novel, solution-processed organic memtransistors (*mem*Ts) with an unconventional redox-gating mechanism are demonstrated. Our synaptic *mem*T device using conjugated polymer thin-film and redox-active solid electrolyte as the gate dielectric can be routinely operated at gate voltages (*V*_GS_) below − 1.5 V, subthreshold-swings (*S*) smaller than 120 mV/dec, and ON/OFF current ratio larger than 10^8^. Large hysteresis in transfer curves depicts the signature of non-volatile resistive switching (RS) property with ON/OFF ratio as high as 10^5^. In addition, our *memT* device also shows many synaptic functions, including the availability of many conducting-states (> 500) that are used for efficient pattern recognition using the simplest neural network simulation model with training and test accuracy higher than 90%. Overall, the presented approach opens a new and promising way to fabricate high-performance artificial synapses and their arrays for the implementation of hardware-oriented neural network.

## Introduction

Since the inception of civilization, researchers have dreamt about building an electronic machine with similar functionality as a brain. As a result, brain structure-inspired neuromorphic computing has been extensively explored as a promising way to realise artificial intelligence systems that are capable of learning and performing complex tasks^1,2^. The first hardware-based neural functionality was demonstrated using Si-based CMOS logic but the demonstrated electronic circuits were cumbersome and consumed huge amount of energy^3^. To date, substantial developments have been made in both software algorithms and hardware designs^4,5^. However, in terms of size, power efficiency, massively parallel connectivity, self-learning capability, fault-tolerant operation, data storage, and data processing, the human brain is still much more efficient than any neuromorphic platform built thus far^6^. In fact, the brain can efficiently process real-time unstructured signals such as sound, light, pressure, heat, and taste, which is impossible to realise using today’s computers. To perform such complex tasks, the brain functions in massively parallel ways using interconnected neurons and synapses. Typically, the action potential signal is passed from one neuron to the next by modulating the chemical fluxes inside the synaptic cleft that changes the synaptic weight in response to the signal frequency^7^. Neurons also emulate the Hebbian learning principle and spike-timing-dependent plasticity (STDP), which forms the basis of learning and memory functions. Notably, the Hebbian postulate states that the synaptic strength among pre- and post-neurons can only be modulated if both are fired momentarily with a mandatory temporal correlation^8^. Mimicking various synaptic functionalities such as excitatory postsynaptic current (EPSC), short-term potentiation and depression (STP/STD), long-term potentiation and depression (LTP/LTD), paired-pulse facilitation (PPF), post-tetanic potentiation (PTP), as well as many others have recently been reported in the literature^6,9,10^. However, reaching the energy efficiency close to the brain (1–100 fJ) for an artificial synaptic event is still very challenging^6,11^ to achieve.

Consequently, many efforts have been devoted to develop low energy consuming synaptic devices and neural circuits using unconventional electronic materials and devices^9^. For example, the demonstration of memristor (MR) in 2008 sparked a strong interest in building neuromorphic circuits using MR^12^. Since then, many memristor designs have been proposed to emulate biological synaptic plasticity, including phase-change memories, resistive switching devices, and so on^13–22^. However, even though these devices consume much less energy compared with the software-driven neural network hardwares, they still cannot achieve the energy efficiency close to the brain. Recently, three-terminal ferroelectric, floating-gate, and electrolyte-gated thin-film transistors (TFTs) based memory devices have been under intense study to mimic synaptic actions^23–25^. In particular, electrolyte gated TFTs have been broadly investigated as their structure is quite similar to that of a biological synapse and they can be operated at low gate voltages (< 3.0 V) due to the large specific capacitance offered by the electrolyte gate dielectrics^26–29^. In these TFTs, the gate electrode resembles a presynaptic input terminal called the axon and the drain electrode corresponds to the postsynaptic output terminal called the dendrite. Accordingly, functionalities of a biological synapse can be simulated by modulating/demodulating the channel conductance (so-called synaptic weight) by applying voltage pulses to the gate (presynaptic) and drain (postsynaptic) terminals. However, the efficacy of a synaptic device depends on several factors including the device structure, used active channel materials, stability of operation, synaptic behaviours, and energy consumption per synaptic event. Hence, exploring and studying alternative materials and device structures is essential to realize high-performance artificial electronic synapses and large-area synaptic arrays.

Here, high-performance, low-voltage redox-gated organic memtransistors (*mem*Ts) which efficiently emulate various synaptic actions of a brain are demonstrated. Thin-film of poly(3-hexylthiophene-2,5-diyl) (P3HT) is used as the channel and redox-active ethyl viologen diperchlorate (EVP) with polyethylene oxide (PEO) solid electrolyte as the gate dielectric. It is shown that the availability of large number of discrete conducting states programmed by voltage pulses applied to the gate and improved transistor performance make *mem*Ts very promising devices to be used as artificial synapses that consume as little energy as 250 pJ per synaptic action (SA). The analog synaptic device concept proposed here can be explored further as the employed organic materials are relatively easy to process and their electronic properties can be tuned as desired using conventional chemical routes and approaches.

## Results and discussion

### Memtransistor characteristics with the multilevel memory property

**Figure 1:**
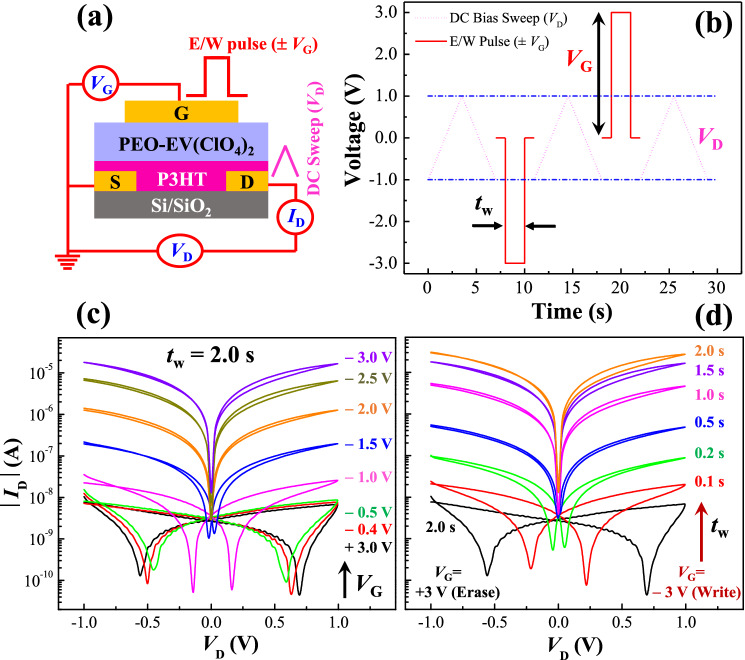
(**a**) Schematic diagram of the redox-electrolyte gated thin-film memtransistor (*mem*T) including the details of the pulse and sweep measurement protocol. (**b**) Schematic representation of the voltage pulses at the gate terminal and DC voltage sweep across source-drain (S-D) contacts to measure the channel conductivity of the fabricated 3-terminal *mem*T devices. (**c, d**) Show multiple discrete conducting states of the device channel measured after applying different voltage pulse amplitudes (*V*_GS_) and widths (*t*_w_), respectively.

An organic memory transistor or memtransistor (*mem*T) is basically a thin-film transistor (TFT) possessing additional resistive switching and memory storage capabilities^30^. Fig. 1a shows the structure of our top-gate bottom-contact organic *mem*T devices using ~ 30 nm thick poly(3-hexylthiophene-2,5-diyl) P3HT thin film as active channel and a ~ 2.5 µm thick drop-cast solid electrolyte film of ethyl viologen diperchlorate ((EV(ClO_4_)_2_) in poly(ethylene oxide) (PEO) as the gate dielectric^26,29^. Typical transistor characteristics reveal that the fabricated *mem*T devices show usual p-channel transistor behaviour with a ON/OFF current ratio >10^8^ and operational voltage |*V*_GS_| or |*V*_G_|< 1.5 V as illustrated in the Supplementary Information Fig. S1. This result also confirms that a suitable negative (− ve) voltage at gate is needed to switch the channel conductivity from a low to a high value. To depict the memory and information storage capability of our *mem*T devices, current–voltage (*I*–*V*) characteristics were recorded to demonstrate the resistive switching (RS) property as per the scheme illustrated in Fig. 1b. Usually, RS property of 3-terminal devices is demonstrated by the channel conductivity switching from a low (OFF) to a high (ON) state by applying a suitable gate voltage (*V*_GS_)^26,31^. As per the scheme, current–voltage (*I*_D_–*V*_DS_) characteristics at the drain were recorded by sweeping the *V*_D_ (or *V*_DS_) in a loop between − 1.0 and + 1.0 V before and after applying suitable gate voltage pulses of amplitude *V*_GS_ and width *t*_w_ as shown in Fig. 1c-d, respectively. Interestingly, multiple conducting states have been observed by varying gate voltage or write pulses from − 0.4 to − 3.0 V in multiple steps keeping the width (*t*_w_) constant at 2.0 s as shown in Fig. 1c. Similarly, multi-level high-conducting (ON) states have been observed just by varying the pulse width *t*_w_ from 0.1 to 2.0 s keeping the write pulse (*V*_G_) amplitude constant at − 3.0 V as shown in Fig. 1d. Moreover, the device shows consistent behaviour of switching back to the low-conducting (OFF) state just by applying an erase (E) voltage bias pulse + 3.0 V, 2.0 s as depicted in both the Fig. 1c-d, respectively. We also observed similar result of consistent RS property for a device fabricated later as illustrated in the Supplementary Information Fig. S2. Calculation shows a minimum energy of 3 nJ (*E* = *V*_GS_ × *I*_G_ × *t*_w_) per switching event considering input pulse − 3.0 V, 0.1 s and gate current 10 nA. Furthermore, this value can be reduced further by optimizing all parameters of energy consumption that is necessary to artificially emulate energy efficient synaptic plasticity as observed in a brain^3^.

Transfer characteristics for the drain voltages (*V*_DS_) starting from − 0.5 to − 2.0 V in step of − 250 mV have also been recorded and it has been found that the value of *V*_DS_ has negligible influence as shown in Supplementary Information Fig. S3a. As such, threshold voltage (*V*_TH_) was calculated at *V*_DS_ =  − 0.5 V and was found to be − 1.87 V (Supplementary Information Fig. S3b)^26^. Here, the observed hysteresis loop area of the transfer curve is crucial to show memory or RS property and is the signature of redox-reactions occurred between the dissociated ethyl viologen (EV^2+^) ions in the dielectric layer and P3HT monomer unit of the polymeric backbone in the active channel^29^. Principally, EV^2+^ is reduced to EV^+^ by accepting an electron and P3HT is oxidized to P3HT^+^ (polaron) by releasing an electron under a suitable negative voltage bias at gate following the chemical equations below^31^.

$$P3HT+{EV}^{+2}\rightleftharpoons {P3HT}^{+}+{EV}^{+},$$

$$EV{\left({\text{ClO}}_{4}\right)}_{2}\rightleftharpoons {EV}^{+2}+2\cdot {\text{ClO}}_{4}^{-}.$$

The minimum potential required for this coupled redox-reaction is found to be about 0.97 V assuming no ohmic losses and considering standard electrode potential (E^0^) values of + 0.52 V ($P3HT\to {P3HT}^{+}$) and − 0.45 V (${EV}^{+2}\to {EV}^{+}$) vs. NHE, respectively^32^. Therefore, the devices need at least − 1.0 V applied to the gate to initiate the redox reaction considering unavoidable ohmic losses for RS based memory application and projected neuromorphic device functionalities. Moreover, the subthreshold swing (*S*) as low as 120 mV/dec was routinely obtained as shown in the inset of Supplementary Information Fig. S3b, which is equal or indeed much smaller *S* than the similar devices reported previously^26,33^. Notably, this low value of *S* indicates that our *mem*T devices are suitable for low-power circuit design of artificial synaptic plasticity and neural functionalities^30^.

### Modulation of synaptic weight by varying training pulse numbers

**Figure 2:**
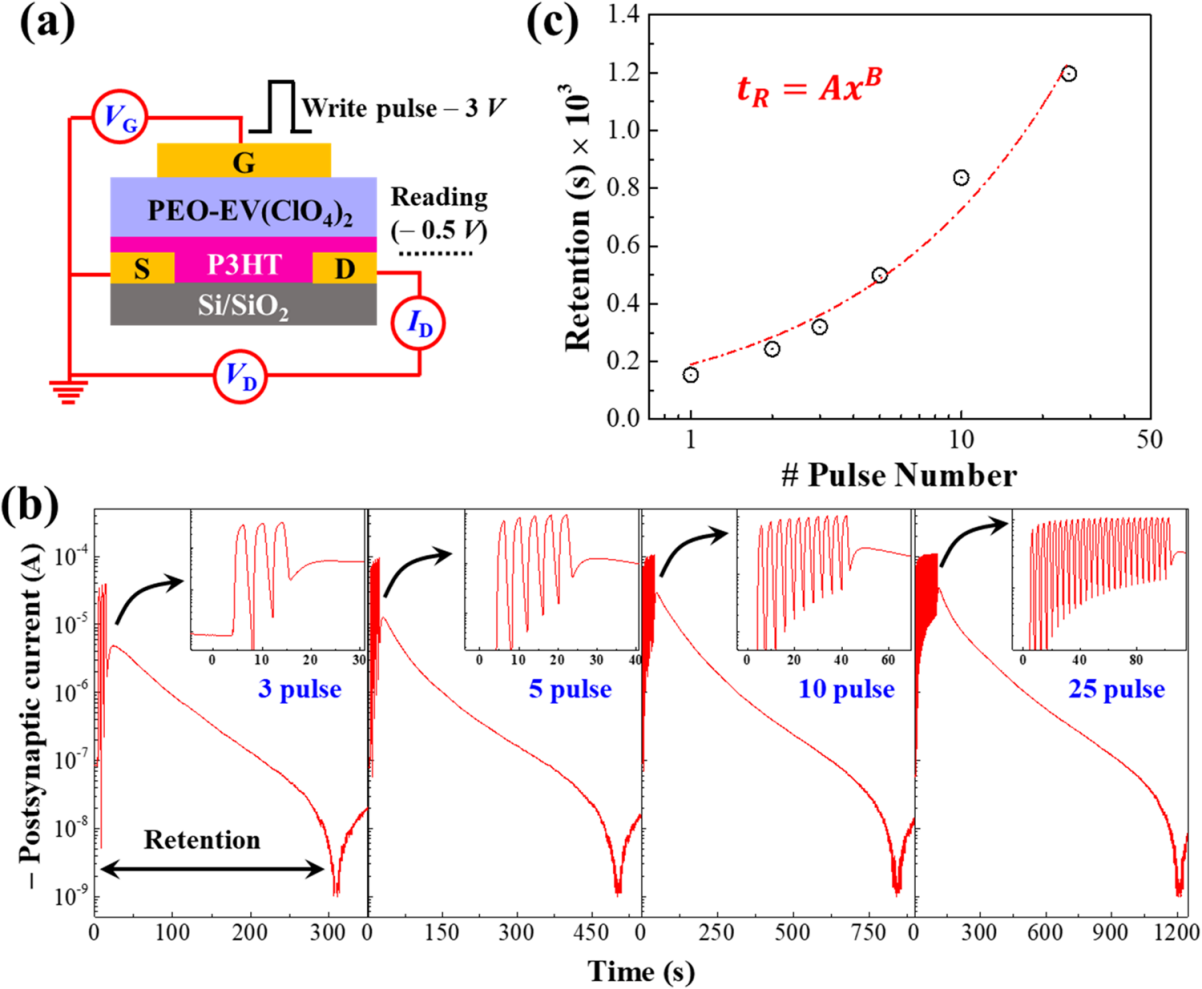
(**a**) Schematic diagram of the *mem*T devices to study EPSC potentiation and retention by varying presynaptic voltage pulse numbers up to 25 pulses. (**b**) Potentiation and retention of EPSC result with increasing number of consecutive presynaptic pulses (− 3.0 V, 2 s) at the gate. (**c**) Dependency of the relaxation time in terms of natural decay of EPSC in open circuit mode to the off-conducting state on number of consecutive pulses applied at the gate.

For any biological neural action, the delay time required to reach the OFF conducting or baseline state after the excitatory postsynaptic current (EPSC) facilitation is crucial to classify the synaptic weight changes in terms of the short-term (STP) or long-term (LTP) potentiation. Fig. 2a represents the testing setup to study the effect of consecutive presynaptic spike numbers on EPSC potentiation and related relaxation time. The consecutive − 3.0 V, 2 s presynaptic pulses were programmed to appear at the gate terminal and the corresponding postsynaptic current at the drain was recorded by applying a voltage bias − 0.5 V until the OFF-conducting state was reached. As illustrated in Fig. 2b, the EPSC responses were monitored temporally by applying 1 to 25 consecutive presynaptic pulses at the gate in open circuit condition (relay-based isolation) to observe the neural decay. We found that the retention for single and dual pulses are of the order of 200 ms which increased further with the applied pulse numbers by following a power law (${t}_{R}=A{x}^{B}$) as illustrated in Fig. 2c. Retention or relaxation time is estimated by the time required for the EPSC response after applying the pulses to reach the current value ~ 1.0 nA through natural decay. Clearly, the result shows that there is at least sixfold enhancement in the retention time as the number of incoming pulses increases from 1 to 25. Interestingly, these results clearly resemble the synaptic plasticity observed in brain that shows the strengthening of synaptic connectivity with the increase of propagation of action potential spikes. Moreover, this response is reflected as the facilitation of memory level, which can be retained for a longer time through training and rehearsal. This is almost similar to the structural model of human brain proposed in 1968 by Atkinson and Shiffrin^34^. Particularly, the model classifies memorization into three separate variants—the sensory register (SR), short-term store, and long-term store. First, the incoming information is stored in the SR for a short while, then it decays, and eventually it is either completely lost or it is transmitted to the short-term store. However, the retention of SR can be improved upon rehearsal and subsequent transformation to short-term store. Then, the information can be transmitted to the long-term store, which can last for few minutes lifetime based on the duration of the rehearsal as observed here for our *mem*T device^34^. Retention around 100 s can be classified as the short-term memory (STM) and higher values of retention represent the long-term memory (LTM).

### Influence of the training voltage pulse anatomy on the synaptic weight change

The temporal retention in the order of 10^3^–10^8^ s has been proposed as one of the highly desired metrics for organic neuromorphic devices^7^. Therefore, investigations to visualize the temporal retention varying pulse anatomy and repetition (rehearsal) were performed which would be useful further for classifying memorization process in a brain. Hence, we have varied presynaptic pulse number (N), width (*t*_w_), and amplitude (*V*_GS_) and simultaneously measured postsynaptic currents for longer time as shown in the Supplementary Information Fig. S4a–c, respectively. Repeated rehearsals using voltage pulse − 3.0 V, 50 ms result in huge variation in retention by increasing number of pulses. We found that the retention time depends much stronger on the number of the applied pulses rather than on the pulse amplitude and width. These results are in strong agreement with our previous reports on the retention times of high-conducting states in *mem*Ts^29,31,35^. However, a minimal amplitude of the presynaptic voltage pulse is necessary to see the prominent potentiation as we observed in a separate test with 50 consecutive spikes and varying amplitude as illustrated in the Supplementary Information Fig. S5. Now, the EPSC response of up to 60 s is considered as a reference value to classify the information storage and processing steps in our synaptic *mem*T device. Typically, EPSC responses below 1 nA are considered as the sensory memory (SM), 1 to 10 nA as STM, and above 10 nA as LTM. Interestingly, rehearsal with 200 pulses results in LTM with estimated retention to be about 85 days (≈ 3 × 10^6^ s) by extrapolating to the EPSC value of 1 nA^7^. Here, all three basic information storage and processing steps, namely SM, STM, and LTM, have successfully been demonstrated as illustrated in the Supplementary Information Fig. S4a^36^. We also observed the similar response in our device just varying number of pulses increasing from 1 to 100 i.e. gradual transformation of synaptic weight from SM to STM and finally to LTM as depicted in the Supplementary Information Fig. S6. Here, STM and LTM are used frequently in neuroscience and represent the same quantities as STP and LTP, respectively in psychology.

**Figure 3:**
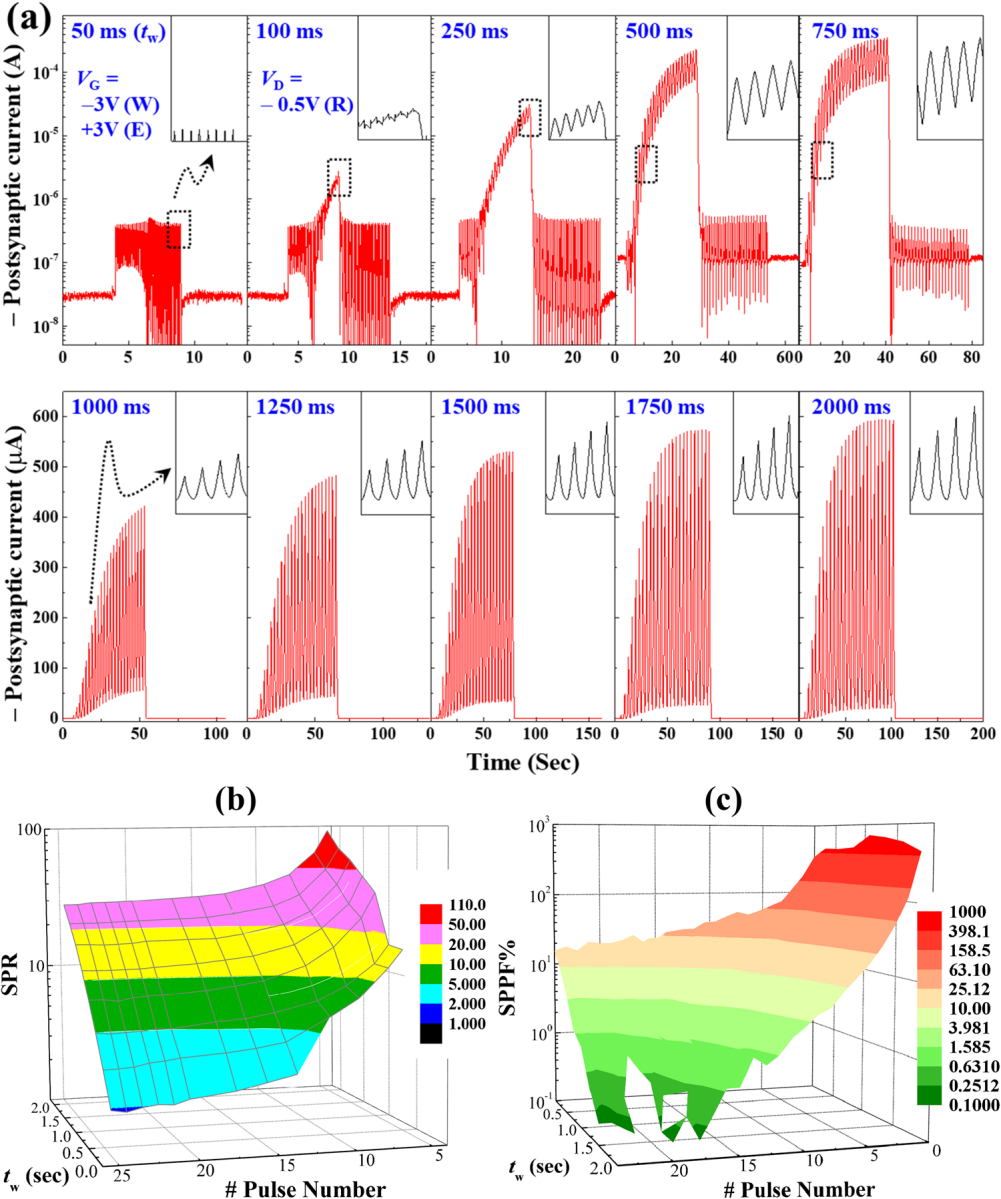
(**a**) Presynaptic pulse width (*t*_w_) dependent variation of the EPSC response to demonstrate spike-width-dependent plasticity (SWDP). 25 write pulses (− 3.0 V) followed by 25 erase pulses (+ 3.0 V) were consecutively applied at the gate by varying *t*_w_ from 50 to 2000 ms. Insets, enlarged portion of the obtained curves to show the potentiation of EPSC for each of the incoming pulses. (**b**) Variation of the synaptic potentiation ratio (SPR) calculated from the two consecutive write (W) pulses for every pulse widths as obtained in (**a**). (**c**) Dependency of the sequential paired-pulse facilitation (SPPF) of synaptic weight on pulse widths and numbers as estimated from the results shown in (**a**).

Availability of at least 100 discrete states is also one of the recommended projection to design efficient organic neuromorphic devices^7^. Consequently, the fabricated *mem*T devices were characterized for resistive random-access memory (ReRAM) applications and we found consistent two-level and multi-level results as shown in the Supplementary Information Fig. S7. Therefore, these *mem*T devices could be investigated further to induce large number of discrete conducting states by varying input voltage pulse parameters at the presynaptic terminal. Hence, the EPSC response was recorded at the drain of our biomimetic synaptic *mem*T device by varying presynaptic pulse width from 50 to 2000 ms as shown in Fig. 3a keeping amplitude constant. In this test, 25 consecutive write pulses (− 3.0 V) followed by 25 erase pulses (+ 3.0 V) were applied at the presynaptic gate terminal for each cases. Results show that the potentiation of EPSC response of our *mem*T device becomes more and more prominent by increasing the width of presynaptic pulses. This is similar to the memorization process through rehearsing many times with longer incoming signals in a brain. In addition, it can also be termed as spike-width-dependent plasticity (SWDP) of an artificial synapse. However, the depression of the synaptic weight here looks very quick upon applying 25 erase pulses and it appears to be almost similar for all measurements in Fig. 3a. Hence, this happens due to the testing protocol in closed circuit configuration as described in the experimental details.

Furthermore, the EPSC response shows an incremental enhancement for every presynaptic pulse that appeared at the gate as highlighted in the insets of all sub-panel of Fig. 3a. This is further investigated through a surface plot by considering both the pulse widths (*t*_w_) and numbers (N) as an independent variable to depict the respective synaptic potentiation ratio (SPR) as shown in Fig. 3b. Here, the SPR is estimated by taking the ratio of postsynaptic high-conducting state for a presynaptic pulse to the premeditated low-conducting state. This result strongly suggests that any point on the surface plot can have the SPR values of up to ~ 10^2^ just by selecting a pair of values of presynaptic pulses (*t*_w_, *N*). It is very significant and resembles the availability of very large number (> 100) of discrete conducting states. In fact, the pulse width and number can be correlated with the stability of incoming information and rehearsal phenomena in a biological brain. This clearly resembles that a stable presynaptic signal initially shows more prominent SPR value i.e., potentiation of synaptic connection or weight and then gradually decreases with further increasing the rehearsal, which is almost similar to the mental fatigue in brain^37,38^. Another quantity similar to paired-pulse facilitation (PPF) is also estimated considering the change in EPSC response for two consecutive pulses in the entire set of 25 pulses as shown in Fig. 3c. This quantity is termed as the sequential paired-pulse facilitation (SPPF) and can be found using the following equation $SPPF\%=\left(\left({S}_{N}-{S}_{N-1}\right)/{S}_{N-1}\right)\times 100\%,$ where *S*_N−1_ and *S*_N_ represent two consecutive EPSC signals. The obtained results show that the SPPF% can also be varied up to about 10^3^. It also emphasizes that a strong facilitation can be observed for fewer rehearsal events and very strong fatigue is observed when rehearsal increases with more incoming signals. Our perception here is that the attained results and corresponding analysis strongly resemble the potentiation of synaptic weight with a very large number of discrete states as it happens in a biological nervous system after receiving various input signals^37,39^.

### Paired pulse facilitation of synaptic weight and memorization in the brain

**Figure 4:**
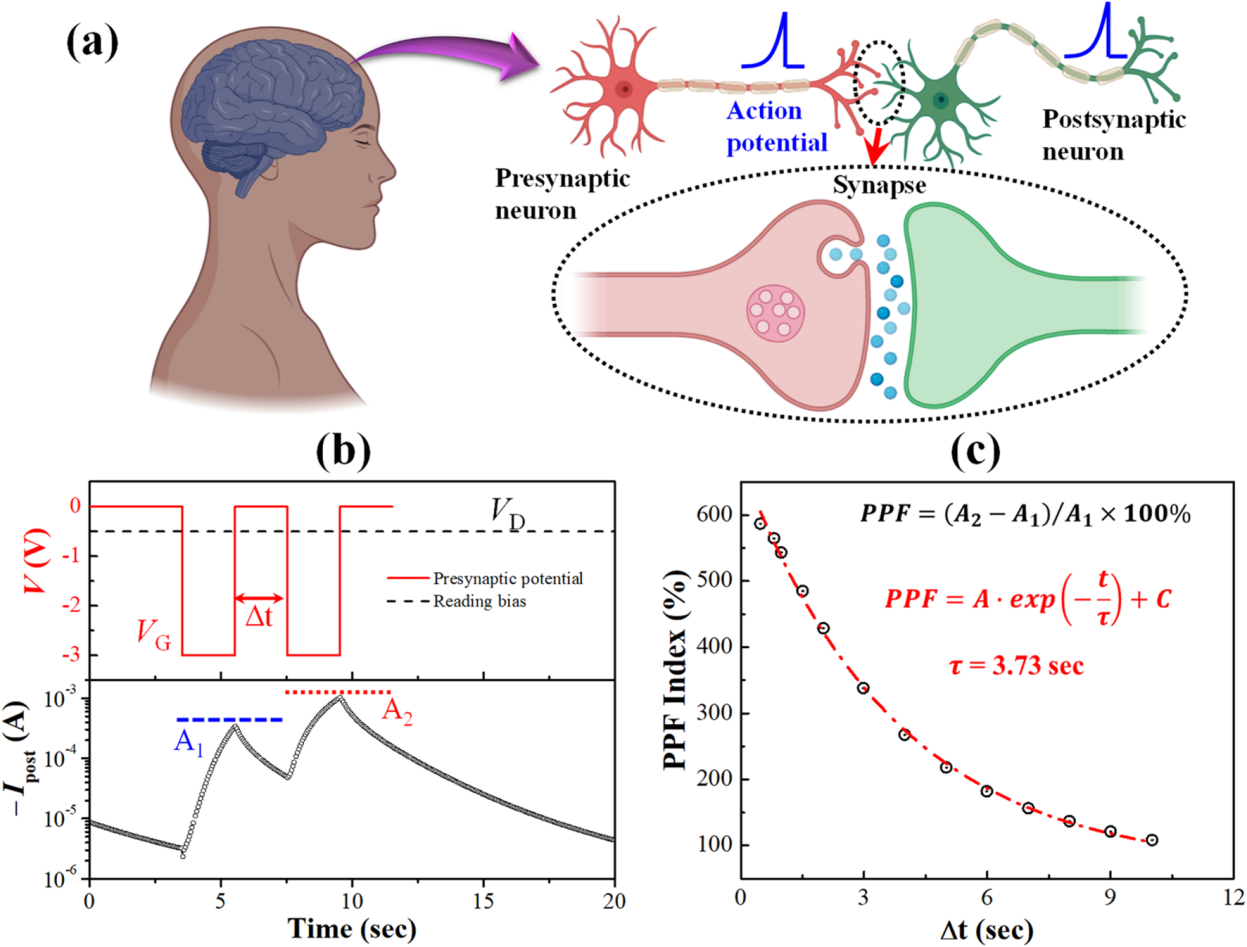
(**a**) Schematic representation of neural connectivity via synapse in a biological brain for the action potential transmission. (**b**) Short-term potentiation by applying a pair of presynaptic spikes (*V*_GS_ =  − 3.0 V) with Δt of 2 s. Postsynaptic currents (*I*_post_) were recorded by biasing the drain at − 0.5 V. (**c**) Paired pulse facilitation (PPF) of the synaptic *mem*T devices obtained by varying the time interval Δt from 100 to 10 s.

In neurons, STM and paired pulse facilitation (PPF) play a very crucial role for any sub-threshold potentiation or strengthening of synaptic weight and these functions are validated in our redox-gated organic *mem*T devices as demonstrated schematically in Fig. 4a. In this testing, two consecutive presynaptic spikes (− 3.0 V, 2 s) with a time gap of Δ*t* are applied at the gate and corresponding EPSC responses were measured at the drain by applying a small voltage bias of − 0.5 V as shown in the top and bottom panels of Fig. 4b, respectively. As a result, EPSC value for the first spike facilitated to a certain peak value of A_1_ and this clearly resembles the strengthening of biological synaptic weight. When a second presynaptic spike appears after a time interval of Δ*t*, the EPSC increases further and reaches to a higher value, A_2_. This is commonly known as paired-pulse facilitation (PPF) of a biological nervous system, which solely depends on the time delay (Δ*t*) between the applied input pulses. Moreover, this is normally demonstrated by calculating the PPF index [$=\left({A}_{2}-{A}_{1}\right)/{A}_{1}\times 100\%$] by varying Δ*t* as shown in Fig. 4c. Significantly, the PPF index result exhibits an excellent fitting with a popular function, called modified Kohlrausch formula [$PPF=A\cdot exp\left(-\frac{t}{\tau }\right)+C$], which is well accepted in psychology to manifest one of the de-memorising functions in brain^40,41^.

### Spiking-timing-dependent plasticity and learning behavior

**Figure 5:**
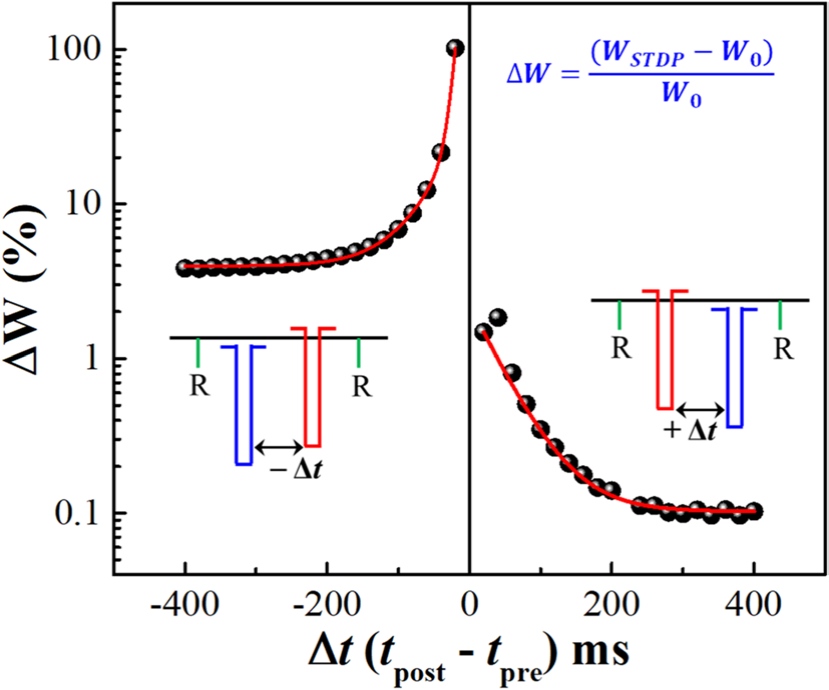
Synaptic weight changes estimated STDP (ΔW %) for both of negative and positive time delays (± Δ*t)* with a postsynaptic read (− 2.0 V, 10 ms) pulse before and after paired pulse propagation. Insets, schematic representation of temporally correlated presynaptic (− 3.0 V, 2 s) and postsynaptic (− 3.0 V, 2 s) pulses for both the time delay of ± Δ*t* to probe STDP responses.

Hebbian learning rule (HLR) is a very basic learning rule to train neural networks but it is very important in validating the performance of artificial electronic synaptic devices. Normally, the most common practice to implement HLR for artificial synapses is to demonstrate the spike-timing-dependent plasticity (STDP). Practically, STDP represents the change in synaptic weight Δ*W* by varying the time delay Δ*t* between the presynaptic and postsynaptic pulses. If presynaptic pulse stimulates momentarily before a postsynaptic pulse, Δ*t* is considered as positive and Δ*W* is also positive, but if a postsynaptic pulse is followed by a presynaptic pulse, Δ*t* is negative and Δ*W* can be either positive or negative^11,42^. Insets of Fig. 5 show the pulses scheme with negative and positive time delay (+ Δ*t*) including the postsynaptic read voltage bias pulses appeared before and after the temporally correlated pulse pair at the pre- and post-neural terminal. In this test, a voltage pulse of − 3.0 V, 2 s is used for both the pre and postsynaptic signal to measure the change in synaptic weight (Δ*W*) and a voltage pulse of − 2.0 V, 10 ms is used to record the EPSC values before (*W*_0_) and after (*W*_STDP_) the pair-pulse transmission event. Then the Δ*W*% for a Δ*t* value is calculated with the help of equation $\Delta W=\left({W}_{STDP}-{W}_{0}\right)/{W}_{0}$ multiplied by 100 only^3^. Finally, changes of Δ*W* % by varying Δ*t* between ± 400 ms were measured and plotted as shown in Fig. 5, which agreeably follows the Hebbian learning rule^43^. Since the synaptic weight change by varying Δ*t* can be fitted with exponential decay functions, it confirms that the STDP behaviour similar to a biological synapse can be achieved with higher efficacy using redox-gated organic memtransistor (*mem*T) devices^44,45^.

### Achieving almost analogous states during potentiation and depression

**Figure 6:**
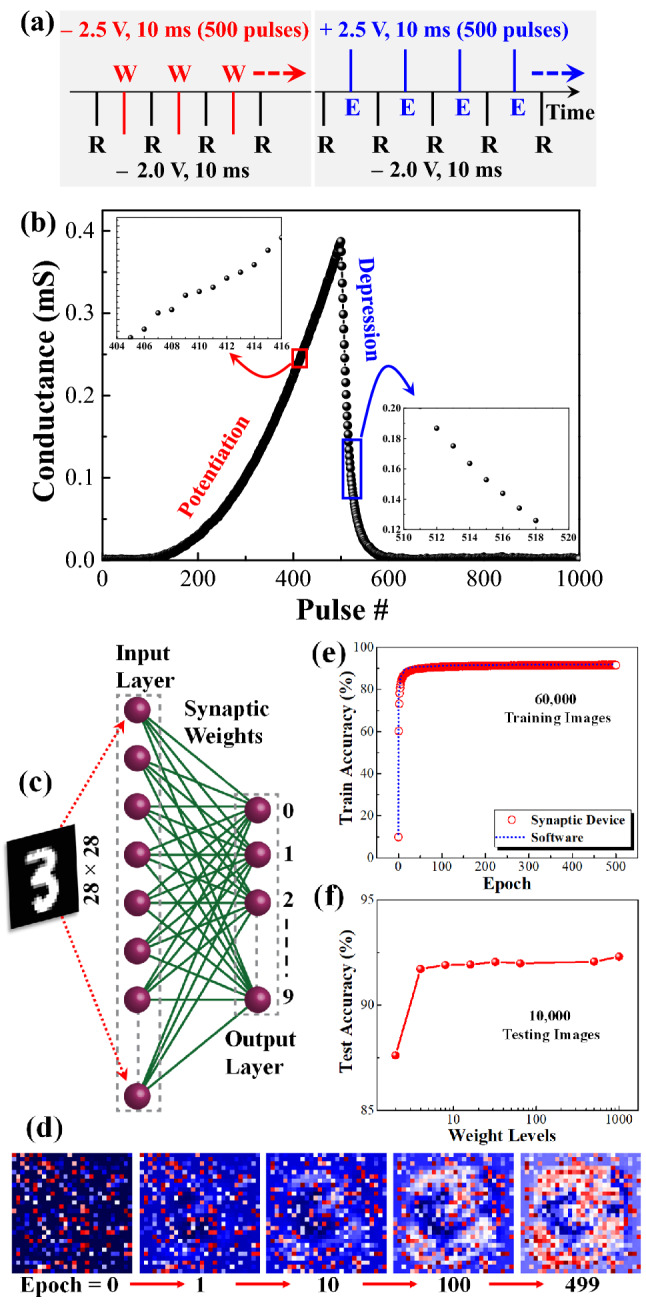
(**a**) Pulsing scheme to demonstrate the excitatory postsynaptic current (EPSC) and inhibitory postsynaptic current (IPSC) response based potentiation (P) and depression (D) of synaptic weight by applying 500 consecutive write (W) pulses (− 2.5 V, 10 ms) and erase (E) pulses (+ 2.5 V, 10 ms), respectively. A read pulse (− 2.0 V, 10 ms) is used to record synaptic weight change after every set of W/E pulses. (**b**) Potentiation and depression curve of synaptic weight change. Insets illustrate enlarged marked areas to visualize discrete conducting states during potentiation and depression. (**c**) Schematic of a single layer neural network used for MNIST pattern recognition. (**d**) Selected weight evolution mapping images from the output neurons layer corresponding to class “3” at different epochs starting from 0 to 499. (**e**) Training accuracy (%) vs number of epochs for both software and actual synaptic device based weight distribution. (**d**) Test accuracy (%) variation with the number or strength of device weight levels.

In brain, neurons and synapses are the crucial components for perception and consequent thought^46^. Normally, neurons are stimulated by external stimuli and produce action potentials, which propagate to the connecting neurons through the synaptic cleft which result in a change in the strength of connection (synaptic weight). This change in synaptic weight could be either excitatory or inhibitory. To establish if our redox-gated organic memT devices posses this functionality, EPSC and inhibitory postsynaptic current (IPSC) response were recorded by applying 500 consecutive presynaptic pulses of − 2.5 V, 10 ms and + 2.5 V, 10 ms, respectively as illustrated with a scheme shown in Fig. 6a. Here, a postsynaptic voltage pulse − 2.0 V, 10 ms was used after every incoming input pulses at the gate to probe the incremental potentiation (P) and depression (D) in terms of EPSC and IPSC response as depicted in Fig. 6b. As shown in Table 1, our organic *mem*Ts display similar performance when compared with the previously reported devices using various electrolytes as the gate dielectric. The result obtained here is almost similar to the learning experience in psychology by the sequentially administered presynaptic excitatory rehearsal with voltage pulses. Similarly, consecutive inhibitory pulses at presynaptic terminal can cause weakening of the synaptic weights that is reflected as loss of memory or depression as evidenced in psychological forgetting. This potentiation and depression result is obtained in our redox-gated *mem*T-based artificial synapse just by consuming energy of 250 pJ per input spike which is definitely a remarkable achievement as this is indeed very close to the desired and recommended metrics for artificial organic synaptic devices^7^. Moreover, this result clearly evidences the capability of our *mem*T devices to continuously modulate the channel conductance to more than 500 discrete conducting states during potentiation and depression which is also much higher than the prerequisite to effectively mimic a synapse and very useful to demonstrate neural network based pattern recognition^7^.

**Table 1:** Performance comparison of the fabricated *mem*Ts with previously reported memory devices using electrolytes as the gate dielectric.

Channel*	Electrolyte* (Phase)	Operation voltage (V)	Energy/SA (pJ)	ON/OFF ratio	Analog states
P3HT^47^	[EMIM][TFSA] + P(VDF-TrFE) (Solid)	7.0			
P3HT^48^	[EMIM][TFSA] + P(VDF-HFP) (Solid)	3.0			
P3HT^49^	[EMIM][TFSA] + P(VDF-HFP) (Gel)	5.0	510		
PTIIG-Np^50^	[EMIM][TFSI] + PS-PMMA-PS (Gel)	2.5			
PEDOT:PTHF^51^	KCl in DI water (Liquid)	1.0			
PEDOT:PSS/PEI^11^	Aqueous NaCl (Liquid)	1.0	10		~ 500
P3HT^52^	PEO/RbAg_4_I_5_ (Solid)	2.0	2		~ 2000
P3HT (This work)	EV(ClO_4_)_2_ + PEO (Solid)	1.5	250	10^5^	> 500

*[EMI][TFSA/I] → 1-Ethyl-3-methylimidazolium bis(trifluoromethylsulfonyl) amide/imide, P(VDF-TrFE) → poly(vinylidene fluoride-co-trifluoroethylene), P(VDF-HFP) → poly(vinylidene fluoride-co-hexafluoropropylene), PTIIG-Np → poly(thienoisoindigo-alt-naphthalene), PMMA → polymethyl methacrylate, PS → polystyrene, PEDOT:PTHF → poly(3,4-ethylenedioxythiophene):poly(tetrahydrofuran), KCl → potassium chloride, NaCl → sodium chloride, PSS → poly(styrenesulfonate), PEI → polyethylenimine, RbAg_4_I_5_ → rubidium silver iodide.

### Efficient pattern recognition using artificial neural network simulation

In order to evaluate the pattern recognition capabilities of the proposed synaptic *memT* devices for neuromorphic hardware implementation, we have simulated a single layer artificial neural network (ANN) without any hidden layers using the normalized conductance values (weights) extracted from the recorded potentiation/depression curve of our synaptic device in Fig. 6b^53,54^. All the simulations were performed using PyTorch package and the detail simulation protocols are demonstrated in the Supplementary Information (Section H)^55^. The well-known Modified National Institute of Standards and Technology (MNIST) dataset is used here for the image recognition task^56^. Each of the (28 × 28) pixel images are linearized to form a (784 × 1) input matrix where each of the pixel values are connected to a neuron in the input layer of the neural network as illustrated with schematic in Fig. 6c. Hence, our simulated neural network has 784 input neurons, number of pixels per label number, and 10 output neurons corresponding to the 10 output classes in the MNIST dataset. We have considered the mean cross entropy loss for the error determination. The as-computed errors are then back propagated for adjusting the weight values for the next iteration. When the full set of training data are passed through the network in several batches, an epoch of training is completed. We have used 500 epochs of training for minimizing the loss function to a low and stable value. Fig. 6d shows the weight evolution images during ANN training using actual synaptic weights obtained from the proposed ST for the output neuron corresponding to digit “3”^57^. It is seen that as the training epochs increase, there is a clear evidence of “learning” observed for the neural network as the corresponding neuron clearly distinguishes between the target label (“3”) from the other digits. Once the training process is completed, we use the test data to compute the pattern recognition accuracy of the network. The entire process (training and testing) has been repeated 5 times for ensuring the reproducibility of the reported data and hence each data point is the mean value for 5 repetitions of the process. In Fig. 6e, we report the training accuracy and mean loss (Supplementary Information Fig. S8) as a function of the number of epochs for both software and synaptic device-based weight distributions. It is clearly observed that the device based (test accuracy = 92.30%) neural network performs similar to the purely software-based (test accuracy = 92.53%) ANN model. This reveals that our synaptic device is highly suited for neuromorphic hardware implementations. Such a high test accuracy for the device based ANN model might be due to the large number of conductance states or weights available for the network training. Further, we have investigated the variation in ANN test accuracy with respect to the weight levels available from the device. This is particularly important from a hardware realization perspective, since many of the conductance levels of the device may not be accessible or stable for cross-bar array implementation. Hence, the performance of the ANN model with the reduced weight levels needs to be studied carefully. From Fig. 6f, we see that the test accuracy for the device is almost constant until about 8 weight levels, below which there is only a marginal drop in test accuracy. This is indicative of the highly robust nature of weight values available from our proposed synaptic transistor for ANN training applications.

## Conclusions

In conclusion, organic memtransistors (*mem*T) that can efficiently mimic the synaptic functionalities as observed in a biological brain are demonstrated. A single *mem*T can serve as a data storage device, as well as as an artificial synapse with various synaptic plasticities, including the EPSC, STP, LTP, PPF, and STDP. The proposed *mem*Ts can also exhibit huge variation in synaptic weight just by tuning the anatomy of incoming presynaptic pulses and increasing rehearsal. In particular, retention of an ON-state is enhanced from 10^3^ to 10^6^ just by increasing repetition or rehearsal from 25 to 200 times even though the pulse width decreased 40 times. Moreover, the presented redox-gated *mem*Ts also performed superiorly as TFTs with ON/OFF current ratios larger than 10^8^, subthreshold swings around 120 mV/dec, and operating voltage *V*_*G*_ <  − 1.5 V. In addition, it was estimated that the energy consumption is as low as 250 pJ per synaptic event that is a significant achievement to mimic synaptic plasticity in a single organic *mem*T device. The energy consumption can be reduced further just by improving the response speed, reducing the input redox-current, and decreasing the threshold voltage as large varieties of organic semiconducting materials are available. Moreover, the ANN simulation result further confirms that our synaptic device performs extremely well for realistic pattern recognition task with a high level of accuracy and reproducibility.

## Methods

### Device fabrication

Redox-gated thin-film transistors were fabricated on the Si/SiO_2_ substrates in a bottom-contact top-gated configuration. The detailed fabrication procedure can be found in reference 25. Briefly, Ti/Au (5/50 nm) source (S) and drain (D) electrodes were formed on the substrates using the standard photolithography and lift-off process. Then, hexamethyldisilazane (HMDS) and 5 mg/ml poly(3-hexylthiophene-2,5-diyl) (P3HT) solutions were spin-coated on the substrates and annealed at 96 ℃ and 140 ℃, respectively. Then, the electrolyte was prepared by mixing equal weight of 4 wt% EV(ClO_4_)_2_ and 5 wt% PEO in acetonitrile and dropcasted in between the S/D electrodes. Finally, 50 nm thick gold (Au) electrode was deposited on top of the electrolyte drop as the top gate contact to complete the fabrication process.

### Electrical characterizations of memTs

Electrical measurements were performed either with a Keithley 4200-SCS parameter analyzer or by combining it with a Keithley 2602B source measurement unit (SMU). Standard transistor characterizations were performed using Keithley 4200. For DC sweep and pulse based memory measurements, a DC sweep voltage was applied between source (S) and drain (D) in dual sweep mode and corresponding drain current (*I*_D_) was measured with the 2602B keeping gate (G) terminal open for initial OFF-conducting state. Then a write pulse voltage (*V*_G_) of width (*t*_w_) was applied at the gate using Keithley 4200 to switch the channel conductivity to the ON state and that was probed by measuring drain current similar to the initial *I–V* recording with Keithley 2602B SMU. To switch back the channel conductivity to the OFF state, an erase voltage pulse was applied to the gate followed by DC *I–V* measurement for probing. This measurement protocol is clearly demonstrated with the results using schematic diagram of device connections and sweep-pulse sequences, as shown in Fig. 1.

### Random access memory (RAM) measurements

The drain current (*I*_D_) was monitored continuously by applying a drain bias voltage − 0.5 V using 2602B SMU. And the ON and OFF states were induced by applying a write (W) pulse − 3.0 V, 2 s and a erase (E) pulse of + 3.0 V, 5 s, respectively, with 5 s wait time between them using a Keithley 4200-SCS. This W/E cycle was repeated for 10 times and corresponding drain current response was recorded as the resistive RAM application test. During the wait time of gate voltage pulses, the gate terminal was kept open or in the floating mode with the help of electrical relay. The schematic diagram of the device connections for performing RAM and the corresponding output is shown in the Supplemental Information Fig. S7.

### Electrical characterizations for synaptic plasticity

Drain current (*I*_D_) or postsynaptic current (PSC) response was measured continuously by biasing the drain terminal with − 0.5 V using the 2602B SMU. 25 presynaptic square voltage pulses of − 3.0 V followed by 25 presynaptic square voltage pulses of + 3.0 V with different pulse width (*t*_w_) starting from 50 to 2000 ms were applied to the gate terminal using 4200-SCS to modulate the channel conductance similar to the synaptic weight or connection strength. Similar measurement protocol was followed for other synaptic tests just varying parameters as required that are explained in the “Results and discussion” section. The time gap between the two presynaptic pulses was varied from 500 ms to 10 s to extract the paired-pulse facilitation (PPF) Index. Schematic of the spike-timing-dependent plasticity (STDP) measurement scheme is demonstrated in the result and discussions section. Initial postsynaptic current response *W*_0_ was measured by applying a read voltage pulse − 2.0 V, 10 ms at the drain. Then a pair of presynaptic (− 3.0 V, 2 s) and postsynaptic (+ 3.0 V, 2 s) voltage pulses with the time delay of ± Δ*t* was applied to the gate terminal followed by the final postsynaptic current response *W*_STDP_ measurement using the same read voltage pulse. The read pulses were applied 10 ms prior to the pre- and postsynaptic pulses and 10 ms after the pre- and postsynaptic pulses to measure *W*_0_ and *W*_STDP,_ respectively.

## Supplementary Information

Supplementary Information.

## Data Availability

Data for the experimental and simulation results are available from the corresponding author upon request.
