# Supplementary material for: Emulation of synaptic functions with low voltage organic memtransistor for hardware oriented neuromorphic computing
Source: Sci Rep. 2022 Mar 9;12:3808. doi: 10.1038/s41598-022-07505-9 (PMC8907356; doi:10.1038/s41598-022-07505-9)
Supplement: Supplementary file 1 — Supplementary Information. [file 41598_2022_7505_MOESM1_ESM.docx]

***Supplementary Information***

**Emulation of Synaptic Functions with Low Voltage Organic Memtransistor for Hardware Oriented Neuromorphic Computing**

*Srikrishna Sagar^1^, Kannan Udaya Mohanan^3^,* *Seongjae Cho^3^, Leszek A. Majewski^2^, and Bikas C. Das^1,*^*

^1^ School of Physics, Indian Institute of Science Education and Research Thiruvananthapuram (IISER TVM), Vithura, Trivandrum, Kerala 695551, India.

^2^ Department of Electrical and Electronic Engineering, University of Manchester, Manchester M13 9PL, UK.

^3^ Department of IT Convergence Engineering, Gachon University, Republic of Korea.

*** Corresponding author**

Email: [bikas@iisertvm.ac.in](mailto:bikas@iisertvm.ac.in)

**ORCID:**

Bikas C. Das: [0000-0002-4750-0542](https://orcid.org/0000-0002-4750-0542)

Leszek A. Majewski: [0000-0001-6544-1286](https://orcid.org/0000-0001-6544-1286)

Srikrishna Sagar: [0000-0002-0121-265X](https://orcid.org/0000-0001-6544-1286)

Seongjae Cho: 0000-0001-8520-718X

1. **Transistor characteristics of *mem*T devices**

Figure S1 represents typical transistor characteristics of the fabricated memT device. Transfer characteristics were recorded by sweeping gate voltage (*V*_G_) starting from +2.0 V to -3.0 V and back to +2.0 V and measuring corresponding drain current (*I*_D_) at a constant drain voltage (*V*_D_) of -0.5 V as shown with a black coloured line. The arrows in the Figure indicate the sweeping direction. The red coloured line shows the gate current (*I*_G_) measured simultaneously with the transfer characteristics as shown in Figure S1(a). Output characteristics were recorded by sweeping the drain voltage (*V*_D_) from 0.2 V to −0.6 V and measuring drain currents (*I*_D_) at different gate voltage (*V*_G_) biases of from −0.5 to −1.5 V in steps of −0.1 V as shown in Figure S1(b).


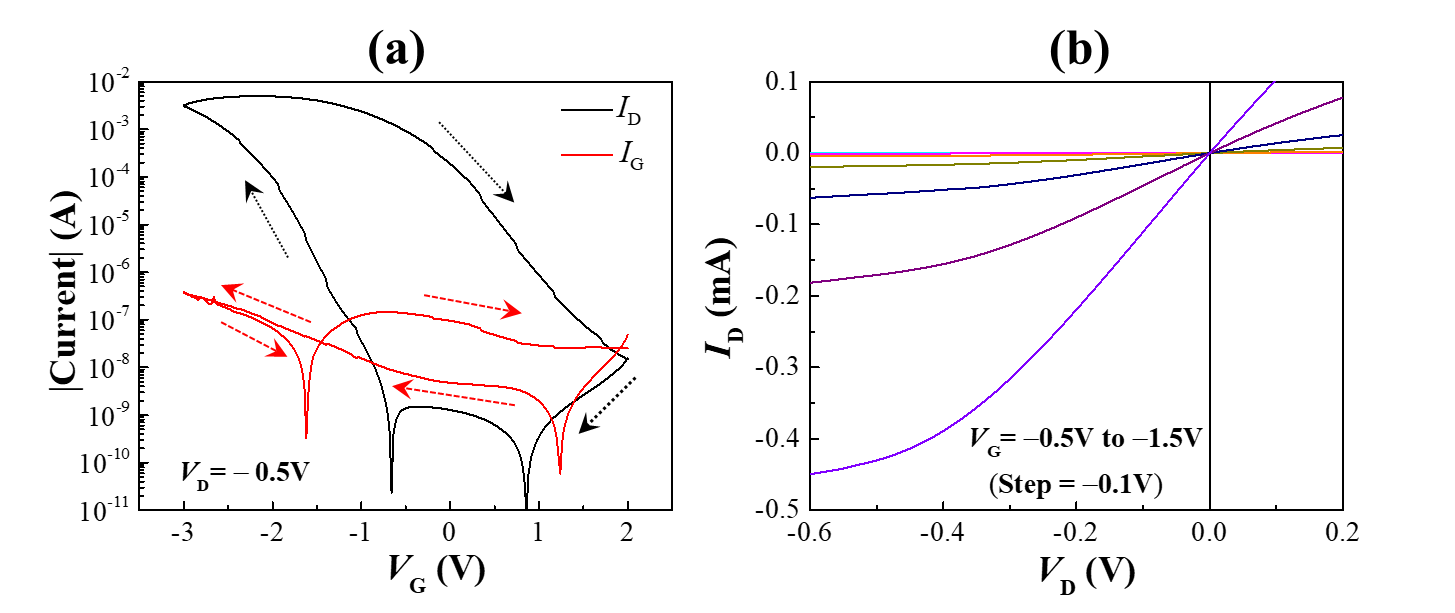


**Figure S1**. Typical transistor characteristics of redox-electrolyte gated organic MemTs. (a) Transfer characteristic (*I*_D_ vs. *V*_GS_) and gate current (*I*_G_ vs. *V*_GS_) curve. (b) Output characteristic (*I*_D_ vs. *V*_DS_) measured as a function of gate voltage (*V*_GS_).

1. **Voltage Pulse Mediated *I-V* Characteristics to Show Resistive Switching**

Resistive switching property of our 3-terminal *mem*T devices was probed by recording current-voltage (*I-V*) characteristics between source (S) and drain (D) before and after applying write (W) voltage pulse -3.0 V, 2 s and erase (E) voltage pulse +3.0 V, 2 s at the gate terminal as shown in Figure S2. This measurement was conducted in the floating mode configuration. Typically, a DC sweep voltage was applied between the source (S) and drain (D) in dual sweep mode from -1.0 V to +1.0 V and back to -1.0 V in step of 20 mV keeping gate terminal open and its corresponding drain current (*I*_D_) was measured. The black line depicts the initial *I-V* characteristics between S-D of the device without applying any pulse voltage at the gate and it shows OFF-conducting state with maximum current ~ -10^-8^ A at -1.0 V. Then a W pulse -3.0 V, 2 s was applied at the gate which actually switched the channel conductance to very high i.e. ON-conducting state with current value about 30 µA at -1.0 V as depicted with red lined *I-V* characteristic in Figure S2. Finally, the channel conductance of our memT device was returned back to the OFF-conducting state by applying a square voltage (*E*) pulse +3.0 V, 2 s at the gate terminal as shown by a blue line in Figure S2 below.

**Figure S2**. Drain *I-V* characteristics without applying voltage pulse (initial, black line), after applying W pulse (red line), and erase (E) pulse (blue line) at the gate terminal of our memT device.

1. **Effect of Drain Voltage on Transfer Characteristics: Threshold voltage (*V*_TH_) and Subthreshold Swing (*S*) Estimation**

Transfer characteristics (*I*_D_ vs *V*_GS_) of our memT device were recorded by varying drain voltage (*V*_D_) starting from -0.50 V to -2.00 V in step of -250 mV as shown in Figure S3(a). Influence of drain voltage found minimal which also reflects as very little change of threshold voltage (*V*_TH_). Therefore, we have estimated *V*_TH_ using transfer curve for *V*_D_ = -0.5 V. Figure S3(b) shows the $\sqrt{\left| I_{D} \right|}vs. V_{G}$ plot and *V*_TH_ extracted from intersection point between the extrapolating linear region and voltage axis. It was found to be -1.87 V, which is exactly matching with our previously reported result on low voltage thin-film transistor (TFT).^[1]^ Inset in Figure S3 (b) shows the log(|*I*_D_|) vs *V*_G_ plot using the same transfer curve for *V*_D_ = -0.5 V, which was used to estimate the subthreshold swing (*SS*) value near the current value of about 1 nA and was found to be 120 mV/dec.


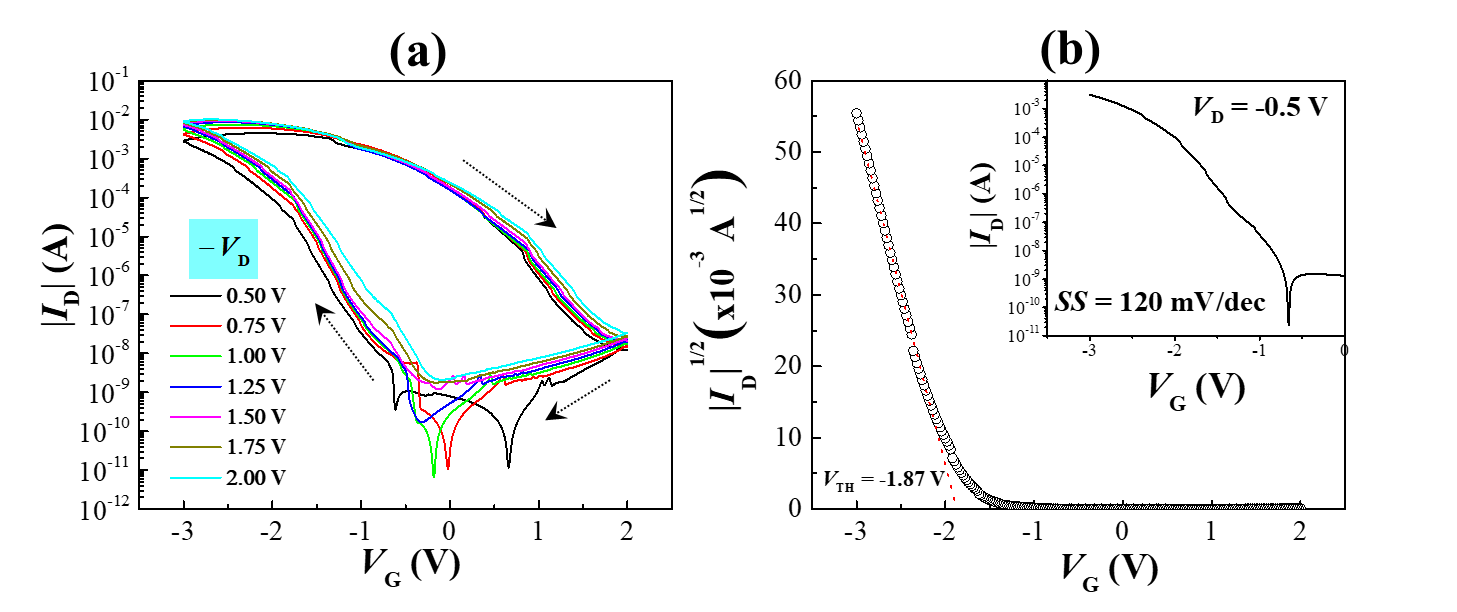


**Figure S3**. (a) Transfer characteristics by varying *V*_D_ of the redox-gated organic memT. (b) $\sqrt{\left| I_{D} \right|}vs. V_{GS}$ plot calculated from the transfer characteristic of *V*_DS_ = -0.5V. Inset in (b) shows log(|*I*_D_|) vs *V*_GS_ of the same transfer curve.

1. **Depicting the Atkinson and Shiffrin Model of Memorization in Brain by Varying Presynaptic Pulse Anatomy**

Figure S4 shows the transformation of sensory memory (SM) to short-term memory (STM) and finally to long-term memory (LTM) of redox-gated organic memT device depending upon the number of rehearsals by presynaptic pulses and its anatomy ( width and amplitude). Drain voltage (*V*_DS_) of -0.5 V was applied continuously to measure the temporal response of the drain current (*I*_D_) in open circuit configuration, i.e., when the presynaptic pulse terminates the gate terminal isolated by a mechanical relay. The number of presynaptic pulses (-3 V, 50 ms) applied at the gate terminal as repetitive rehearsal by varying numbers from 1 to 200. As the number of rehearsal increased, the memT reached to the higher conducting states with longer retention times as shown in Figure S4(a). When the number of rehearsals was less than five, memT device showed SM (high conducting state dropped to initial state instantaneously as soon as the gate pulse was removed). When the number of rehearsals was increased to 20, the device memorization transformed to STM with data retention for a longer period close to 100 s. Finally, data retention have increased a lot with presynaptic pulse numbers from 50 to 200, which clearly indicate the transition of memorization process from STM to LTM. Extrapolating data for 200 pulses shows retention of the order of 10^6^ s. We further tested our device with a constant presynaptic pulse number of 20 just varying pulse width and amplitude as shown in Figure S4 (b and c), respectively. It is observed that the device shows only SM and STM by rehearsing with 20 pulses for all the parameters used, which clearly indicate that rehearsal with presynaptic pulses is very important to train our memT device as per Atkinson and Shiffrin model.^[2]^


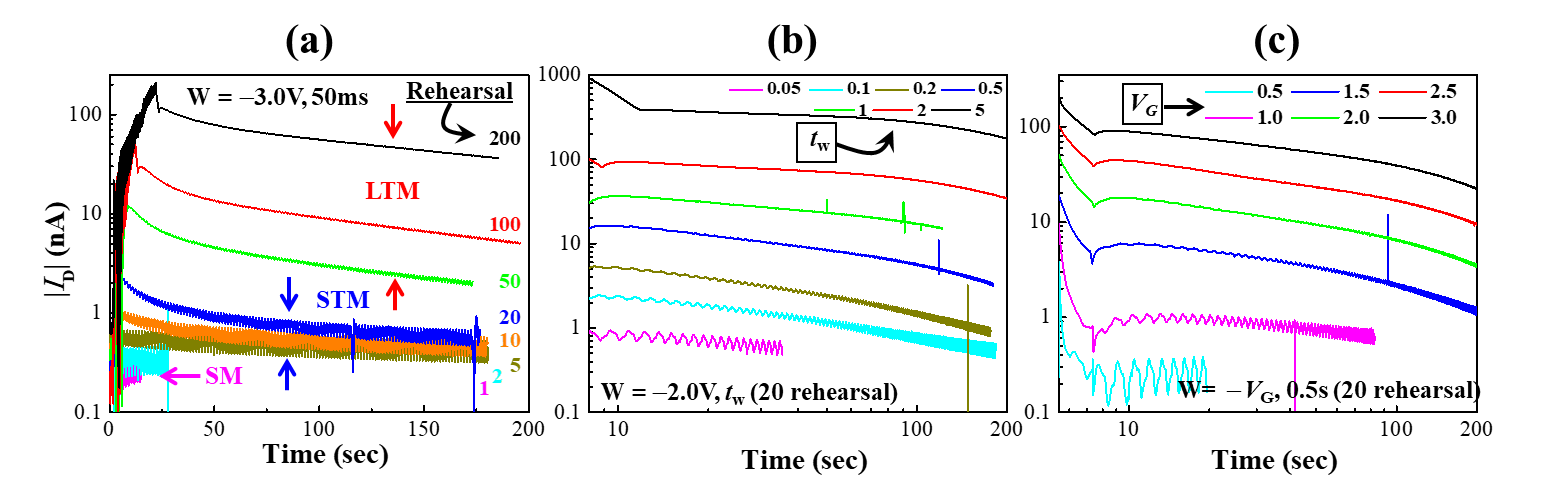


**Figure S4**. Temporal response ($\left| I_{D} \right|$ vs *t*) were recorded by varying presynaptic pulse number from 1 to 200 (a), widths from 50 ms to 5 s (b), and amplitudes from − 0.5 V to − 3.0 (c).

1. **Voltage Pulse Amplitude Dependent Potentiation**

Effect of presynaptic voltage pulse amplitude on synaptic potentiation was also investigated by measuring EPSC response at different voltage amplitudes for 50 repetition as shown in Figure S5. The inset of the left panel of Figure S5 shows the device schematic and necessary electrical connections. Keeping pulse width (*t*_w_) constant to 2 s, consecutive 50 presynaptic pulses of different amplitude were applied and corresponding drain current (*I*_D_) as EPSC response was captured by applying a postsynaptic drain bias voltage (*V*_D_) of -0.5 V. When the presynaptic voltage is -1.0 V, there was virtually no change in the synaptic weight (drain current) of the memT device and -1.5 V produces minimal response as shown in Figure S5 left and middle panel, respectively. When the gate voltage (*V*_G_) amplitude was increased to -2.0 V, the device channel conductivity enhances largely similar to the potentiation of synaptic connection and weight as shown in the right panel of Figure S5.


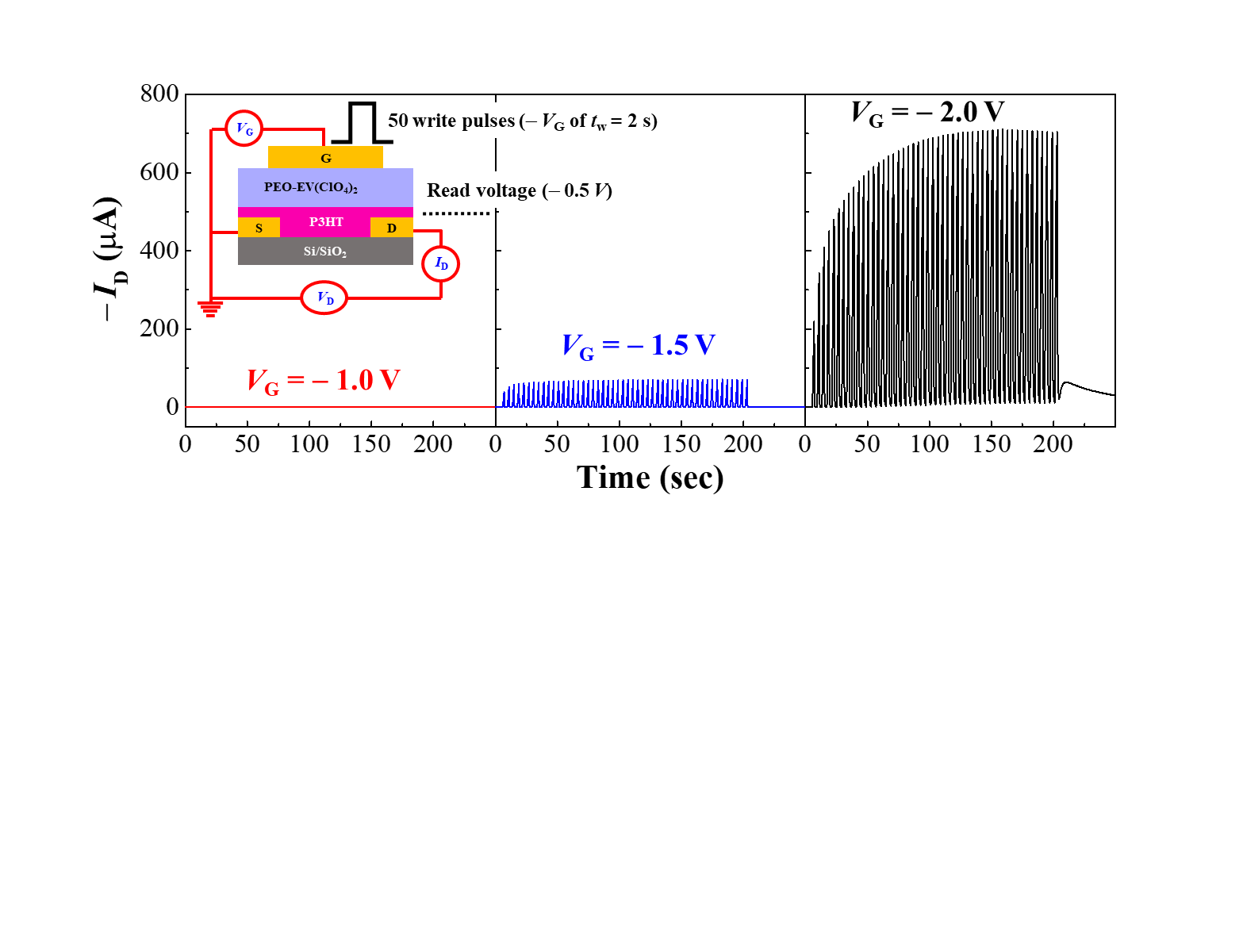


**Figure S5**. Presynaptic voltage pulse amplitude dependent synaptic weight change for the redox-gated organic memT device. Consecutive 50 pulses of amplitudes −1.0 V (left panel, red), −1.5 V (middle panel, blue), and −2.0 V (right panel, black) having a constant width (*t*_w_) of 2 s were used in this test. Temporal postsynaptic currents were recorded with a drain voltage of − 0.5 V.

1. **Pulse Number Dependent Synaptic Plasticity**

Now the effect of presynaptic voltage pulse numbers on the excitatory postsynaptic current (EPSC) response was also investigated as shown in Figure S6. We used a presynaptic pulse −2.5 V, 250 ms for this test by varying pulse numbers starting from 1 to 100 in closed circuit condition (maintaining gate voltage bias at 0 V) as shown in Figure S6(a). A postsynaptic reading drain voltage bias −0.5 V was used to record EPSC responses. The EPSC response remains weak when very few pulses from 1 to 10 appeared at the gate, which looks very similar to the sensory memory (SM) effect of the biological nervous system as depicted in Figure S6 (b). Then the response gradually transformed to intermediate short-term memory (STM) states by repeating spikes 25 and 50 times as the EPSC values are still being facilitated as illustrated in Figure S6(c).^[3-4]^ Finally, the EPSC is further facilitated to a saturation current value of ~100 µA after receiving 100 spikes consecutively as shown in Figure S6 (d), which appears to closely follow the assumption made by Atkinson and Shiffrin on memorization process in brain through repeated rehearsal process.^[2]^ Moreover, the EPSC result in this test shows gradual and consistent potentiation for every pulse that appears at the presynaptic terminal, which is clearly visible in Figures S6 (b, c, and d). It is also noticeable here that the EPSC quickly decays back to the initial OFF-state immediately after stopping the incoming pulses to the gate. This decay is a result of the measurements which were performed in a closed-circuit configuration.

**Figure S6.** Presynaptic voltage pulse number dependent potentiation of synaptic weight. A presynaptic voltage pulse −2.5 V, 250 ms is used in this test by just varying repetition number from 1 to 100.

1. **Resistive Random-Access Memory (ReRAM) Applications of memT**

Figure S7 (a) shows the device schematic along with electrical connections for the read/write/read/erase (R/W/E/R) programming scheme to test the random access memory (RAM) behaviour.^[5-6]^ Figure S7 (b) shows the drain current (*I*_D_) response to ten cycles of write (W) and erase (E) pulses with *V*_G_ = ±3 V to the gate terminal. Here, red and blue markers indicate the high (ON) and low (OFF) conducting states, respectively. To record the drain current (*I*_D_), a continuous DC read (R) bias voltage of −0.5 V was applied at the drain terminal.

Here, the device is operated in floating mode keeping the gate terminal in the open circuit configuration after applying the W/E voltage pulses. As shown in Figure S7 (b), the channel current jumps to *I*_D_ ≈ 50 µA (ON-state) after “pumping” with W pulses and returns to *I*_D_ ≈ 0.5 nA (OFF-state) after applying E pulses resulting in a high ON/OFF current ratio of ~10^5^ between the two extreme conducting states. Figure S7 (c) shows the highly reproducible four distinctive high-conducting (ON) states demonstrating multiple level resistive switching for multi-level random access memory (ReRAM) applications in response to the four different write pulses. Write pulse applied at the gate terminal was varied from -1.5 to -3 V in step of -0.5 V. This strongly suggests that the presented memTs can be used to produce large number of discrete conductivity states just by optimizing the W and/or E pulse parameters and this is one of the very important as well as recommended metrics to emulate biological synaptic functions efficiently.^[7]^


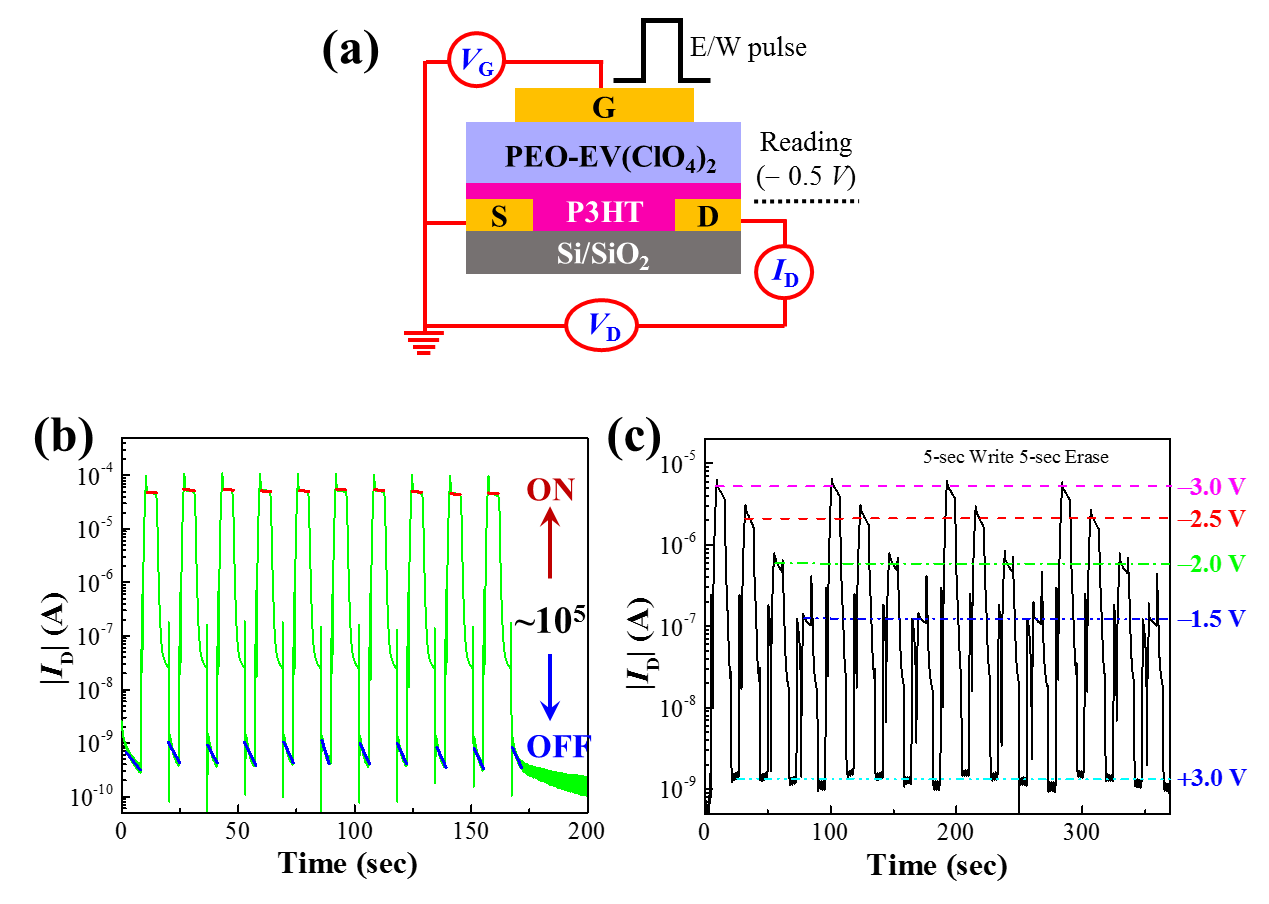


**Figure S7.** (a) Schematic diagram for ReRAM measurements of the redox-gated organic memT device. (b and c) Temporal drain current (*I*_D_) response for the binary and multi-level ReRAM application, respectively.

1. **Neural Network Simulation Protocol and Mean Loss During Training**

Here, we have employed the well-known Modified National Institute of Standards and Technology (MNIST) dataset for the image recognition task. The MNIST dataset consists of a total of 70,000 grayscale images of 28 x 28 pixel handwritten digits ranging from “0” to “9”. The dataset is further divided into 60,000 training images and 10,000 testing images for our neural network simulation. Each of the 28 x 28 pixel images are linearized to form a 784 x 1 input matrix where each of the pixel values are connected to one neuron in the input layer of the neural network, as shown schematically in main manuscript Figure 6 (c). Hence, our simulated neural network has 784 input neurons and 10 output neurons corresponding to the 10 output classes in the MNIST dataset. The input values are then multiplied with the corresponding weight values and summed up to give $y_{j}=\sum_{i,j} w_{ij}x_{i}$where *y_j_* denote the input to the *j*^th^ output neuron, *w_ij_* correspond to the synaptic weight between *i*^th^ input neuron and j^th^ output neuron, *x_i_* represents the value at the i^th^ input neuron. Each of the values *y_j_* undergo non-linear transformation through the rectified linear unit (ReLU) activation function. Once the output is obtained at the output neurons, the loss function is used to calculate the error between the target value and the predicted value.

**Figure S8.** Mean loss vs. number of epochs during training for both the purely software-driven and the synaptic device-based weight distributions.

**REFERENCES**

[1] S. Sagar, A. Dey, B. C. Das, *ACS Appl. Electron. Mater.* **2019**, 1, 2314.

[2] R. C. Atkinson, R. M. Shiffrin, in *Psychology of Learning and Motivation*, Vol. 2 (Eds: K. W. Spence, J. T. Spence), Academic Press **1968**, p. 89.

[3] M. Korte, D. Schmitz, *Physiol Rev* **2016**, 96, 647.

[4] T. Ohno, T. Hasegawa, T. Tsuruoka, K. Terabe, J. K. Gimzewski, M. Aono, *Nat Mater* **2011**, 10, 591.

[5] R. Kumar, R. G. Pillai, N. Pekas, Y. L. Wu, R. L. McCreery, *J Am Chem Soc* **2012**, 134, 14869.

[6] B. C. Das, R. G. Pillai, Y. L. Wu, R. L. McCreery, *ACS Appl. Mater. Inter.* **2013**, 5, 11052.

[7] Y. van de Burgt, A. Melianas, S. T. Keene, G. Malliaras, A. Salleo, *Nat Electron* **2018**, 1, 386.
